# Diverse *Xylaria* in the Ecuadorian Amazon and their mode of wood degradation

**DOI:** 10.1186/s40529-023-00403-x

**Published:** 2023-10-25

**Authors:** Nickolas N. Rajtar, Joshua C. Kielsmeier-Cook, Benjamin W. Held, Cristina E. Toapanta-Alban, Maria E. Ordonez, Charles W. Barnes, Robert A. Blanchette

**Affiliations:** 1 Present Address: Department of Plant Pathology, University of Minnesota, St. Paul, MN 55108 USA; 2 QCAM Fungarium, Pontificia Universidad Católica del Ecuador, Quito, Ecuador; 3 Present Address: Forest Health Protection-Region 5, USDA Forest Service, San Bernardino, CA 92408 USA; 4 Present Address: Department of Microbiology and Plant Pathology, University of California, Riverside, 92521 USA

**Keywords:** Xylariales, Ecuador, Fungi, Wood decay, Tropical rainforest

## Abstract

**Background:**

*Xylaria* is a diverse and ecologically important genus in the Ascomycota. This paper describes the xylariaceous fungi present in an Ecuadorian Amazon Rainforest and investigates the decay potential of selected *Xylaria* species. Fungi were collected at Yasuní National Park, Ecuador during two collection trips to a single hectare plot divided into a 10-m by 10-m grid, providing 121 collection points. All *Xylaria* fruiting bodies found within a 1.2-m radius of each grid point were collected. Dried fruiting bodies were used for culturing and the internal transcribed spacer region was sequenced to identify *Xylaria* samples to species level. Agar microcosms were used to assess the decay potential of three selected species, two unknown species referred to as *Xylaria* 1 and *Xylaria* 2 and *Xylaria curta*, on four different types of wood from trees growing in Ecuador including balsa (*Ochroma pyramidale*), melina (*Gmelina arborea*), saman (*Samanea saman*), and moral (*Chlorophora tinctoria*). ANOVA and post-hoc comparisons were used to test for differences in biomass lost between wood blocks inoculated with *Xylaria* and uninoculated control blocks. Scanning electron micrographs of transverse sections of each wood and assay fungus were used to assess the type of degradation present.

**Results:**

210 *Xylaria* collections were sequenced, with 106 collections belonging to 60 taxa that were unknown species, all with less than 97% match to NCBI reference sequences. *Xylaria* with sequence matches of 97% or greater included *X*. aff. *comosa* (28 isolates), *X. cuneata* (9 isolates) *X. curta* and *X*. *oligotoma* (7 isolates), and *X*. *apiculta* (6 isolates)., All *Xylaria* species tested were able to cause type 1 or type 2 soft rot degradation in the four wood types and significant biomass loss was observed compared to the uninoculated controls. Balsa and melina woods had the greatest amount of biomass loss, with as much as 60% and 25% lost, respectively, compared to the controls.

**Conclusions:**

X*ylaria* species were found in extraordinary abundance in the Ecuadorian rainforest studied. Our study demonstrated that the *Xylaria* species tested can cause a soft rot type of wood decay and with the significant amount of biomass loss that occurred within a short incubation time, it indicates these fungi likely play a significant role in nutrient cycling in the Amazonian rainforest.

**Supplementary Information:**

The online version contains supplementary material available at 10.1186/s40529-023-00403-x.

## Background

The Ascomycota are a large taxonomic group of fungi and many genera, such as *Xylaria* are important components of forest ecosystems. The biology and ecology of most xylariaceous fungi are not well known (Rogers 1979). *Xylaria* are an extremely diverse genus of fungi. Many can colonize wood and cause a soft rot type of attack that degrades cellulose and hemicellulose and modifies and degrades some lignin (Nilsson et al. 1989). Soft rot is a form of decay that was first found in aquatic environments (Duncan 1960; Findlay 1984) but recent investigations have shown that fungi causing soft rot in wood are also often found in many other environments including dry and wet terrestrial sites, Polar Regions and in other extreme environments (Blanchette 2000; Blanchette et al. 1994, 2004, 2010). Fungi in the *Xylariaceae* family are common in tropical rainforests throughout the world. Much of the research on them has focused on their taxonomy and relatively little is known about their ecological activities in different environments (Chacon and Gonzalez 2019; Dennis 1956; Rogers 2000; Van der Gucht 1995; Whalley 1996). A recent publication has identified a location in the Ecuadorian Andean cloud forest as being incredibly diverse in Xylariales (Vandegrift et al. 2023). Yasuní National Park, located in the Ecuadorian Amazon, is one of the most biodiverse places in the world and has an extraordinarily large species richness of trees, animals, and insects (Bass et al. 2010). Our observations at Yasuní National Park suggest that fungi, and the *Xylariaceae*, are also exceedingly abundant and diverse at this location. Although a few studies have identified some of the taxa present (Guevara et al. 2018; Laessøe and Lodge 1994), more comprehensive studies are needed to better understand the diversity of these fungi and to elucidate their role in ecosystem functioning in tropical rainforests.

*Xylaria* species have been studied as far back as 1799 and for centuries since have been identified by taxonomists and mycologists around the world (Dennis 1956; Hsieh et al. 2010). Many studies have documented the distribution of *Xylaria* globally and to date, *Xylaria* has been reported on six of the seven continents (Dennis 1956, 1958; Greenhalgh and Chesters 1968; Ju and Rogers 1999; Rogers 2000; Rogers and Samuels 1986; San Martin Gonzalez and Rogers 1989; Whalley 1996) with several reports of *Xylaria* in the Amazon region (Laessøe & Lodge 1994; Vandegrift et al. 2023; Vega Gutierrez et al. 2020). At Yasuní National Park, several cosmopolitan species of fungi have been identified with most of the species being in the family *Polyporaceae* (Toapanta Albán, 2014). Other recent studies identified some unusual Basidiomycota in Yasuní National Park (Toapanta Albán et al. 2021, 2022). These species produce aerial melanized rhizomorphs to facilitate colonization of substrates and better survive in the very wet environment of the rainforest. Many *Xylaria* also produce melanized pseudosclerotial plates that completely enclose substrates. These structures appear to aid in protecting the fungus from excessive moisture or drying and to prevent antagonism and displacement by other microorganisms, as has been demonstrated for other fungi producing pseudosclerotial plates (McDougall and Blanchette 1996; Rizzo et al. 1992; Toapanta Albán et al. 2021).

Soft rot is a unique form of wood decay that differs from other forms of wood degradation such as white and brown rot (Blanchette 2000; Eriksson et al. 1990; Savory 1954). Soft rot is produced by Ascomycota and is characterized by the degradation of woody secondary cell walls causing chains of biconical and cylindrical cavities (Type 1 soft rot) within the secondary cell wall. Another type, referred to as Type 2 soft rot, erodes the cell wall from the lumen towards the middle lamella but leaves the middle lamella relatively unaltered (Blanchette 1995; Eriksson et al. 1990). Since the characteristics of this type of decay are not commonly known, illustrative examples of Type 1 and Type 2 soft rot are presented in the supplemental information for this manuscript (Additional file 1). The decay produced by *Xylariaceae* found in the Amazon rainforests, especially Yasuní National Park in Ecuador, has not been investigated.

It is well documented that trees that are high in various extractives containing polyphenolic compounds are able to tolerate harsh environmental factors (Hillis 1987, 1999; Kirker et al. 2013; Taylor et al. 2002). Most woods from trees in the tropics are known to be extractive-rich and appear to resist brown and white rot fungal decay to varying degrees (Bultman and Southwell 1976; Carneiro et al. 2009). There has been some documentation of *Xylariaceae* species causing degradation and mass loss of tropical woods but knowledge is lacking on the degradation mechanism for this important group of fungi as compared to other fungi from temperate areas of the world (Nilsson et al. 1989; Pointing et al. 2003; Whalley 1996).

In this research paper, we report on the identity of xylariaceous fungi collected at Yasuní National Park using molecular methods and sequencing the ITS region of rDNA. We also report on the decay potential of three different isolates of *Xylaria* selected from our investigations using wood from four different species of trees with varying densities and extractives. These studies provide a better understanding of the biology and ecology of the *Xylariaceae* present in the Ecuadorian Amazon Rainforest and suggest an important role for these fungi in nutrient recycling of woody biomass at this location and in other tropical forests throughout the world.

## Materials and methods

Fungi were collected during two separate trips to Yasuní National Park, Ecuador (0° 41′ 0.5″ S 76° 23′ 58.9″ W) in a one-hectare plot divided into a 10-m by 10-m grid. All *Xylaria* fruiting bodies within a 1.2-m radius of each grid point (N = 121) were collected for further processing (https://forestgeo.si.edu/sites/neotropics/yasuni). The study area was selected based on field plots identified by ecologists studying plant diversity (Loses and Egbert 2004). Research was carried out under a QCAM Fungarium research permit of the Pontificia Universidad Católica del Ecuador (MAE-DNB-CM-2015-0039). Fungal samples were processed and deposited in the QCAM Fungarium in Quito.

At the sampling locations, all *Xylariaceae* encountered were collected from trees, leaf litter or from decaying wood and labeled with a unique identifier, including the trip year and grid point number. The fungal material collected was dried and desiccated in a large drying oven at Yasuní Research Station. Culture isolations were made using Petri dishes containing acidified malt extract agar amended with streptomycin sulfate (AMA +) (15 g of Difco Bacto-agar, 15 g of Difco Bacto malt extract per liter of deionized water, 0.1 g streptomycin sulfate and 2 ml 85% lactic acid added after autoclaving). Isolates were grown out at 21 °C for 14 days and then transferred to another petri dish containing malt extract agar (MEA) (15 g Difco Bacto malt extract, 15 g Difco Bacto agar and 1 L deionized water) to obtain pure cultures. DNA extraction was performed with mycelia from pure cultures using a NaOH extraction protocol according to (Osmundson et al. 2013). Fresh fungal material for each collection was also preserved in dimethyl sulfoxide (DMSO) solution and used for DNA extraction (Dawson et al. 1998).The rDNA of the internal transcribed spacer region (ITS), using primers ITS1F and ITS4 (Gardes and Bruns 1993), was amplified via a polymerase chain reaction (PCR) according to previous studies (Blanchette et al. 2016; Held et al. 2021). Gel electrophoresis was done using SYBR green I pre-stain and read with a Dark Reader DR45 to confirm the successful PCR reactions. Sanger sequencing was completed in both directions, and consensus sequences were assembled using Geneious Prime 2023.1.2 https://www.geneious.com. The NCBI BLASTn algorithm was used to compare sequences to known taxa in Genbank. All sequences are reported in Table 2 and sequences that had a score of 97% or higher and matched to a taxonomic publication or type specimen were identified to species level in Table 2.

Three *Xylaria* species (two unknown species referred to as *Xylaria* 1 (OR052384) and *Xylaria* 2 (OR052385) and *Xylaria* 3 referred to as (*Xylaria curta)* (OR052386) were selected for microcosm studies to determine their capacity to cause wood decay. The three *Xylaria* species were selected based on how frequently the strains had been isolated, how quickly the cultures grew, and if they produced a large amount of mycelium in culture. Cultures used for the decay experiments were incubated at 22 °C for 14 days. Agar microcosms were used to assess the decay potential of the *Xylaria* isolates following methods adapted from Toapanta et al. (Toapanta Albán et al. 2022). Wood from four species of tropical trees were selected based on their varying heartwood densities and extractives (Table 1). Wood used for the study was obtained from sawmills in Quito, Ecuador. The selected wood was balsa (*Ochroma pyramidale*), melina (*Gmelina arborea*), saman (*Samanea saman*) and moral (*Chlorophora tinctoria*). The balsa and melina wood were cut into wood blocks 2 × 2 × 2 cm and the saman and morel wood were cut into 1 × 1 × 1 cm. Each block was labeled sequentially and then oven-dried at 95 °C for 48 h, removed from the oven, and allowed to cool in a desiccation chamber. Dry weight was recorded as W1 (time 0). The wood blocks were rehydrated by submerging them in deionized water for 24 h, sterilized by being placed in a single layer (6 blocks or less per dish) in glass Petri dishes and autoclaved for 60 min twice at 121 °C. Blocks were cooled to room temperature and promptly placed in microcosms.

**Table 1:** Wood used in the decay study with common Ecuadorian name, name scientific name, and density of heartwood

	Common Name	Scientific Name	Density (g/cm^3^)
1	Balsa	*Ochroma pyramidale*	0.04
2	Melina	*Gmelina arborea*	0.58
3	Saman	*Samanea saman*	0.72–0.88
4	Moral	*Chlorophora tinctoria*	0.89

**Fig. 1:**
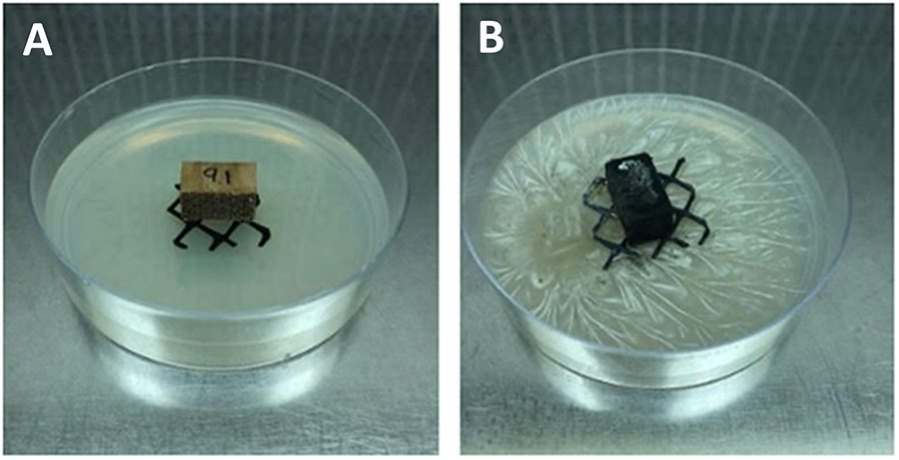
Agar microcosm decay studies. **A** Uninoculated wood block and **B** inoculated blocks after 110 days

Agar microcosms contained a minimal nutrient media consisting of 1.5 g NH_4_NO_3_, 2.5 g KH_2_PO_4_, 2 g K_2_HPO_4_, 1 g MgSO_4_-7H_2_O, 2.5 g glucose, and 0.1 g thiamine per liter that had been previously used to evaluate soft rot fungal decay in other studies (Abrams 1948; Held and Blanchette 2017; Worrall et al. 1991). Microcosms were set up using deep (100 × 25 mm) plastic petri dishes with 15mL of low nutrient agar and a sterile piece of plastic mesh placed in the center of the plate, that was autoclaved twice at 121 °C for 60 min prior to use in the study. A small square of inoculum (5 × 5 mm^3^) was added to the agar plate and allowed to grow out for 14 days. This was done with all three *Xylaria* isolates, using 10 replications for each fungus / wood type. For the controls, wood blocks were treated similarly but a non-inoculated (no fungus) piece of agar was used. In the case of the moral wood control, only 8 blocks were used due to the availability of the wood. Wood blocks were placed on the plastic mesh and plates were sealed with parafilm. Microcosms were stored in plastic containers and incubated at room temperature 22 °C for 110 days (Fig. 1).

The wood blocks were removed from petri dishes after 110 days of incubation at 22 °C. The mycelium was gently removed from each block with a sterile scalpel and the blocks were then dried in an oven at 100 °C for 48 h. Blocks were cooled in a desiccation chamber and dry weight were recorded as W2. Mass loss was calculated as ((W1 − W2)/ W1) × 100. Where W1 was the initial dry weight of each block and W2 was the final weight for each block. The percent weight loss representing biomass lost, for each type of wood caused by each fungus, was recorded. Two wood blocks for each treatment were not dried but frozen at -20 °C and prepared for micromorphological studies.

### Micromorphological studies

Fungal decay was observed by taking inoculated and uninoculated wood samples, cutting them into small segments to prepare for scanning electron microscopy, following previous methods used by Held et al. (2017). Samples were placed in 24-well plates and infiltrated with 50% Tissue-Freezing Medium (Cat. # TFM-5, General Data, Cincinnati, OH, USA) under vacuum, for 1–2 min. Samples were then frozen at − 20 °C and sectioned in a cryostat freezing microtome. A transverse cut was made to obtain a clean surface for examination with scanning electron microscopy. Cut samples were thawed, rinsed in de-ionized water, and air-dried. They were then mounted on aluminum stubs with carbon adhesive tape and coated with gold/palladium (3 nm thickness), using a sputter coater (Cressington 108auto, Ted Pella INC., Redding, CA, USA). Observations of wood cell wall degradation and sound wood were made with a Hitachi S35N scanning electron microscope.

### Statistical analysis

An analysis of variance (ANOVA) was used to test for differences in mean percent weight loss between the control and each assay fungus within each different wood type. The Tukey–Kramer method was used to adjust family-wise error rates during simultaneous comparisons of all pairwise differences in mean percent weight loss between different treatments. Statistical significance was set at alpha of 0.05 for both ANOVA and the Tukey–Kramer method. All analyses were performed in R version 4.1.3 R Core Team (2023). R: A language and environment for statistical computing. R Foundation for Statistical Computing, Vienna, Austria. https://www.R-project.org/.

## Results

**Fig. 2:**
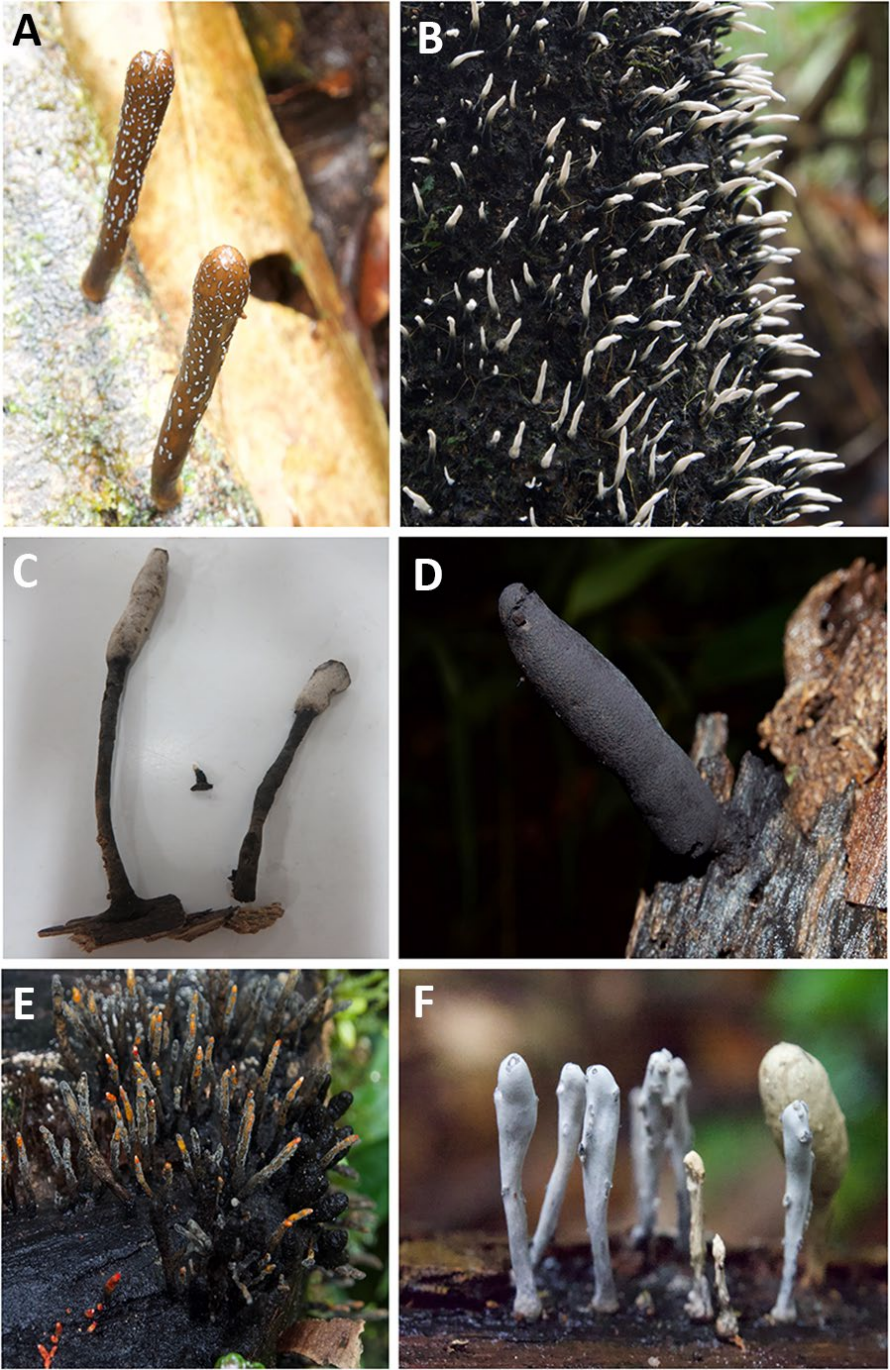
Diverse *Xylaria* from Yasuní National Park. Three of the fungi used in the decay study were *Xylaria* 2 (**A**), *Xylaria* 1 (**B**), and *Xylaria curta* (**C**). **D**–**F** Show other diverse forms of Xylaria

A diverse group of *Xylaria* was found during the study with different morphological characteristics (Fig. 2). Identification based on sequencing of the ITS region of the *Xylaria* sampled and the number of samples belonging to each taxon are presented in Table 2. Out of 210 *Xylaria* collections sequenced, the most frequent *Xylaria* taxa isolated (106 collections belonging to 60 taxa with 106 different operational taxonomic units (OTUs)) were classified as just *Xylaria* sp. since they matched sequences on NCBI megablast search at less than 97%. Identifying these isolates further will require additional taxonomic analyses. Other *Xylaria* with sequence matches of 97% or greater were *X*. aff. *comosa* (28 isolates), *X. cuneata* (9 isolates), *X.* *curta* and *X*. *oligotoma* (7 isolates), and *X*. *apiculta* (6 isolates). A consensus sequence was obtained for each sample by aligning forward and reverse sequences and used for BLAST searches. Four collections thought to be *Xylaria* were found to match only to the family *Xylariaceae* and 3 were in other non-Xylariaceae families. All fungi and their GenBank accession numbers are listed in Additional file 2.

**Table 2:** Isolates identified as *Xylaria* species and closely related taxa based on DNA ITS sequencing

Species Name of *Xylaria*	Number of Samples
*Xylaria* sp.	106
*Xylaria* aff. *comosa*	28
*Xylaria cuneata*	9
*Xylaria curta*	7
*Xylaria oligotoma*	7
*Xylaria apiculata*	6
*Xylaria enterogena*	5
*Xylaria cocophora*	4
*Xylaria cristata*	4
*Xylaria ophiopoda*	4
*Xylaria xanthinovelutina*	4
*Xylaria ianthinovelutina*	3
*Xylaria meliacearum*	3
*Xylaria multiplex*	3
*Xylaria schweinitzii*	3
*Xylaria globosa*	2
*Xylaria longipes*	2
*Xylaria plebeja*	2
*Xylaria arbuscula*	1
Non *Xylaria*	7
Total	210

**Fig. 3:**
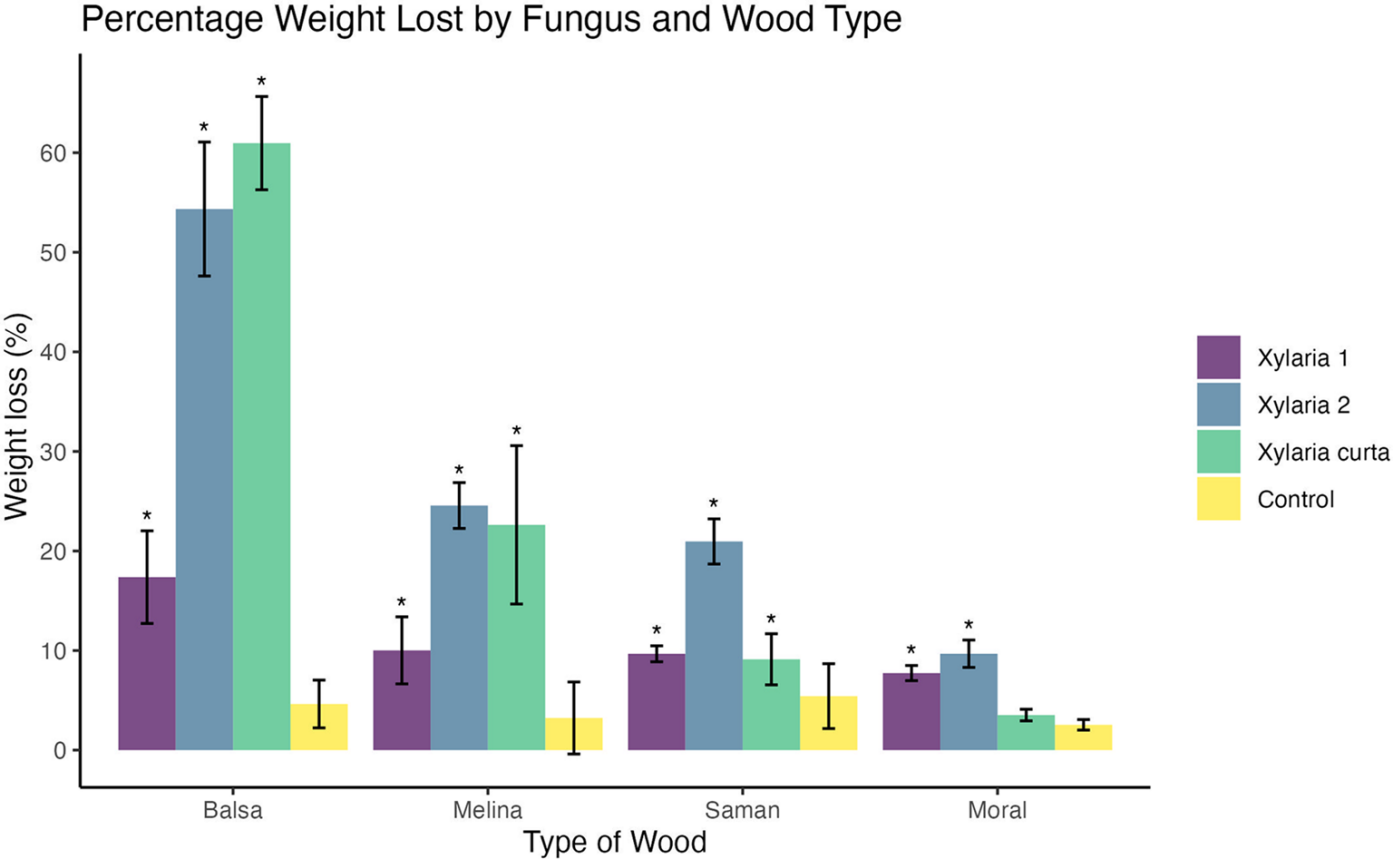
Bar graph showing the percent of weight loss for each wood after inoculation and incubation for 110 days. Controls were uninoculated wood blocks

All *Xylaria* species tested were able to cause degradation in the four wood types. Significant biomass loss was observed compared to the uninoculated controls, and balsa and melina woods had the greatest amount of biomass lost overall. *Xylaria* 2 caused the greatest degradation and biomass loss in all the woods tested followed by *Xylaria curta*. For balsa and melina wood, the percent biomass lost was greatest when decayed by *Xylaria curta* and *Xylaria* 2*,* respectively. *Xylaria* 2 also had the greatest effect on biomass lost in saman and moral woods. *Xylaria* 1 also had significant biomass loss (Fig. 3).

In general, the amount of degradation caused by the *Xylaria* isolates tested was significantly different from the uninoculated controls with the exception of *Xylaria curta* treatment compared to the control using moral wood. ANOVA analysis of treatments using balsa wood, showed significant differences among them (ANOVA, F = 321.3, 3 df, p < 0.001). *Xylaria curta* resulted in the highest biomass loss at 61%, followed by *Xylaria* 2 (54.3%), and *Xylaria* 1 (17.3%). Significant differences among treatments in melina wood (ANOVA, F = 45.9, 3 df, p < 0.001) were also observed. *Xylaria* 2 resulted in the highest biomass loss at 24.5%, followed by *Xylaria curta* (22.6%), and *Xylaria* 1 (10.0%). The differences in percent biomass loss compared to control, uninoculated wood were statistically significant for each inoculated treatment (*Xylaria* 2 p < 0.001; *Xylaria curta* p < 0.001; *Xylaria* 1 p = 0.01). In saman wood, ANOVA analysis showed significant differences among treatments (ANOVA, F = 78.7, 3 df, p < 0.001). *Xylaria* 2 resulted in the highest biomass loss at 21.0%, followed by *Xylaria* 1 (9.7%), and *Xylaria curta* (9.1%). The percent of biomass lost in each inoculated treatment was significantly different from the control (*Xylaria* 2 p = 0.006; *Xylaria curta* p < 0.001; *Xylaria* 1 p = 0.002). Analysis of biomass loss in moral wood also showed some significant differences among treatments (ANOVA, F = 139.1, 3 df, p < 0.001). However, only *Xylaria* 2 and *Xylaria* 1 differed significantly from the control. *Xylaria* 2 had the greatest biomass loss at 9.7% followed by *Xylaria* 1 at 7.7%. Both differed significantly from the control (p < 0.001). All statistical tables can be found in Additional files 3 and 4.

### Micromorphological studies

**Fig. 4:**
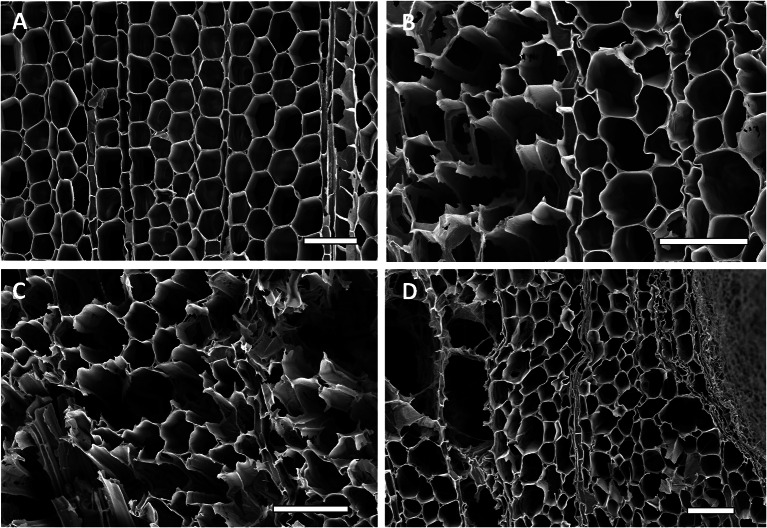
Scanning electron micrographs of transverse sections of balsa wood (*Ochroma pyramidale*) uninoculated and inoculated with three different species of *Xylaria.* **A** Uninoculated balsa wood showing sound cell walls displaying the morphological characteristics of the intact wood, **B**–**D** Wood inoculated with *Xylaria curta* (**B**), *Xylaria* 2 (**C**) and *Xylaria* 1 (**D**) Removal of cell wall material was observed and all three *Xylaria* isolates caused a Type 2 soft rot. Cell walls were eroded to varying degrees and thinned from the lumen towards the middle lamella. The middle lamella remained relatively unaffected except were voids formed from the exceedingly weaken cell walls. Bar, 100 µm (**A**–**D**)

**Fig. 5:**
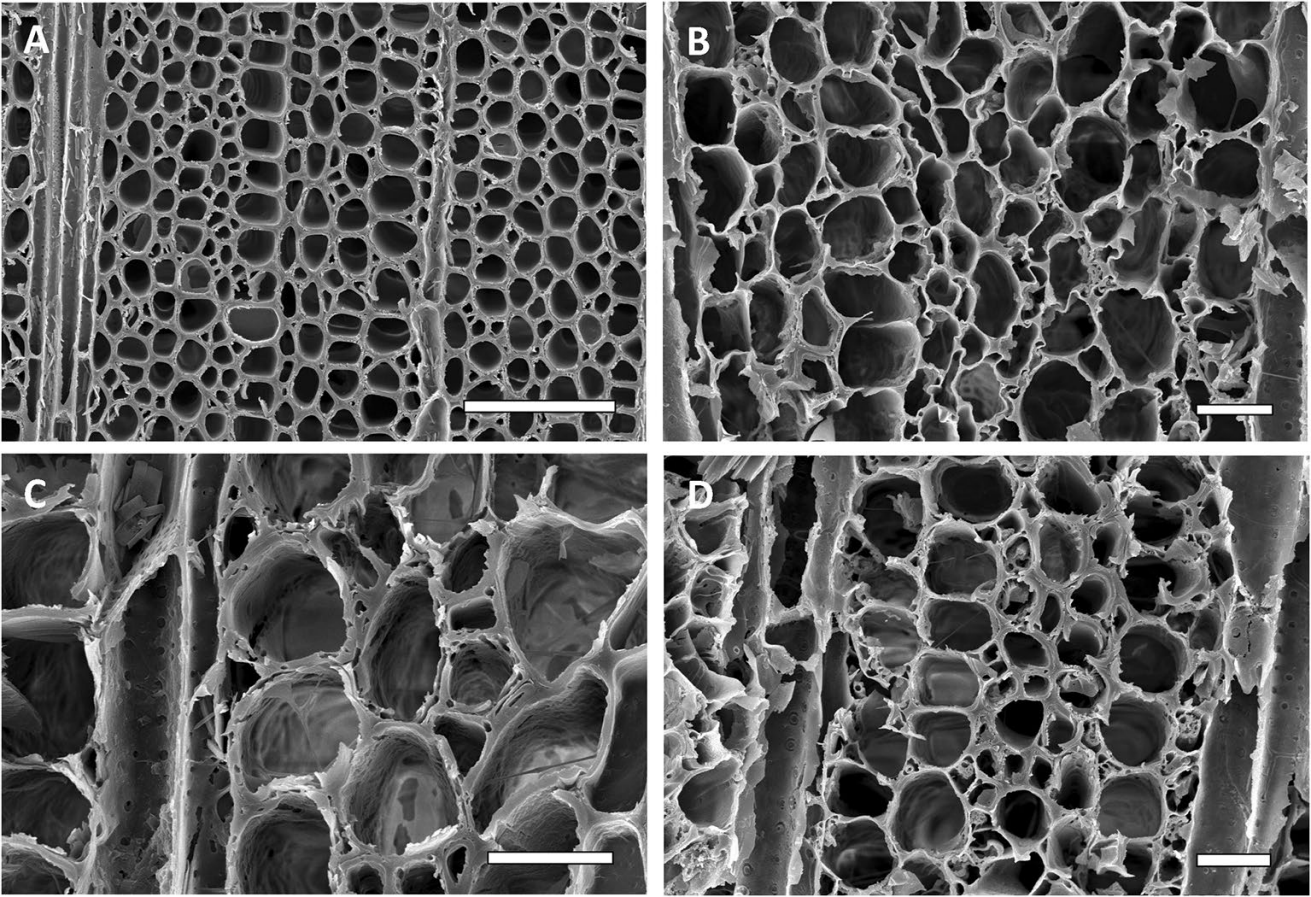
Scanning electron micrographs of transverse sections of melina (*Gmelina arborea*) wood uninoculated and inoculated with three different species of *Xylaria.* **A** Uninoculated melina wood showing all cell walls and components intact, B to D *(Xylaria curta* (**B**), *Xylaria* 2 (**C**) and *Xylaria* 1 (**D**). Type 1 and Type 2 soft rot were evident. Type 2 soft rot can be seen in B with the cell walls almost completely eroded from the lumen towards the middle lamella. Some cells also have Type 1 soft rot with cavities evident in some cell walls. Soft rot cavities were also present in wood decayed by *Xylaria* 2 (**C**) and *Xylaria* 1 (**D**). Bar 100 µm (**A**) and 25 µm in **B**–**D**

**Fig. 6:**
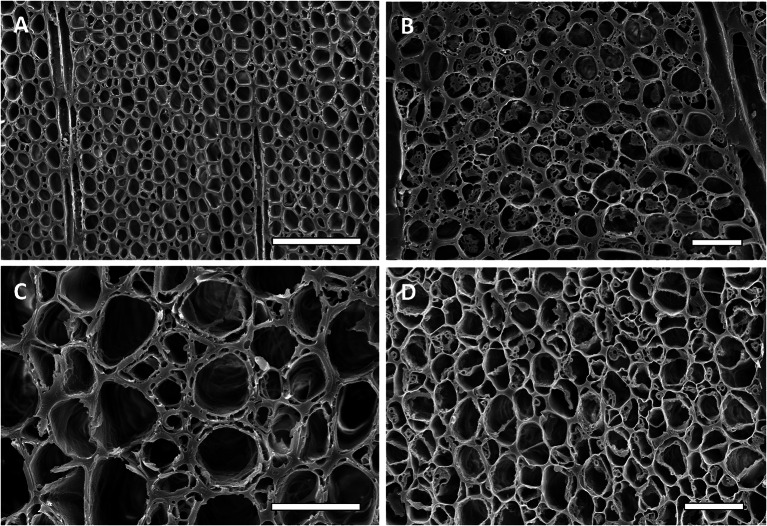
Scanning electron micrographs of transverse sections of saman (*Samanea saman*) wood uninoculated and inoculated with three different species of *Xylaria.* **A** Uninoculated saman wood showing sound cell walls, **B**–**D** Wood inoculated with *Xylaria curta* (**B**), *Xylaria* 2 (**C**) and *Xylaria* 1 (**D**). Type 1 soft rot attack is present in cells of saman wood with all three *Xylaria* isolates. B and C show moderate amounts of degradation with some coalescing of cavities in secondary walls. Some cells had extensive cavity formation, but the middle lamella remained intact. Erosion of secondary walls (Type 2 soft rot attack) and Type 1 cavity formation can be seen in **D**. Bar = 100 µm in **A**, 25µm in **B**–**D**

**Fig. 7:**
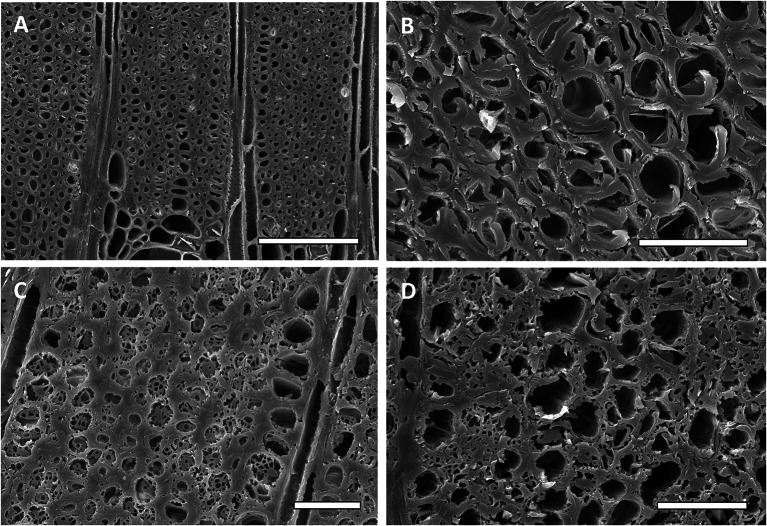
Scanning electron micrographs of transverse sections of moral (*Chlorophora tinctoria*) wood uninoculated and inoculated with three different species of *Xylaria.* **A**) Uninoculated moral wood showing all cell walls that are intact with thick fiber cell walls (**B**–**D**) Wood inoculated with *Xylaria curta* (**B**) *Xylaria* 2 (**C**) and *Xylaria* 1 (**D**). All inoculated woods had Type 1 soft rot attack with cavities present in the secondary walls. Advanced stages of Type 1 soft rot are evident in the thick fiber cell walls of wood (**C** and **D**). Bar = 100 µum in A, and 25 µm in **B**–**D**

Balsa and melina woods, the two woods tested that had lower density, had cell wall erosion typical of Type 2 soft rot fungi. Secondary cell walls were eroded and thinned from the lumina of cells toward the middle lamella. In some areas of the decayed wood, advanced stages of erosion and complete degradation of the secondary walls were evident. The middle lamella, however, was not degraded. These two types of wood had the greatest biomass loss as compared to the other two woods, saman and moral (Fig. 3). There was also a large amount of cell wall material removed by each fungus present (Fig. 4). In Fig. 4, the balsa wood cells show thinning of the cell walls that weakened cells resulting in some voids (B and D). *Xylaria curta* and *Xylaria* 2 caused the greatest mass loss in this type of wood with over 60% of mass loss caused by *Xylaria curta* and over 50% mass loss caused by *Xylaria* 2 (Fig. 3). Melina wood (the second least dense wood used in our study) showed more damage to the S_2_ portion of the cell wall (Fig. 5B and D). There were also mycelia present in some cells. Thinning of the cell walls make the cells appear enlarged with the loss of the secondary wall. Mass loss was greater by *Xylaria* 2 followed by *Xylaria curta* with both fungi causing more than 20% mass loss to the melina wood. Type 1 soft rot was more prevalent in the denser saman and moral wood. Cavities were present throughout the thick secondary walls of fiber cells (Figs. 6, 7).

## Discussion

This study shows that *Xylaria* found in the Ecuadorian rainforest plots studied are remarkably diverse and abundant. This amount of diversity is consistent with other reports of Xylariales in Ecuador (Vandegrift et al. 2023). These fungi have also been found in abundance in other tropical rainforests throughout the world. *X. curta* has also been reported in the United States, India, and China (Ma and Li 2017; Ramesh 2012; Rogers 1983). Another species, *X. oligotoma* has been reported in Papua New Guinea and Malaysia (Dennis 1974; Van der Gucht 1995). Although previous investigations some of these *Xylaria* species indicate that they may be common throughout the tropics, Yasuní National Park in Ecuador appears to have enormous diversity (Valencia et al. 2004). Since tree species richness at this site has been found to be among the greatest in the world, the study reported here strongly suggests that *Xylaria* species diversity may also be among the richest in the world. These fungi also appear to play a key role in the decomposition and carbon cycling in the rainforest.

The endophytic nature of many *Xylaria* and their ability to become established in specific tree species may be responsible for the abundance of diversity (Chen et al. 2013). The endophytic stage may also serve as a strategy for *Xylaria* to rapidly colonize substrates once the branch, limb, or entire tree dies, reducing competition in resource capture from faster growing species. Having endophytic colonization ability may also aid survival in adverse or extreme conditions (Rukachaisirikul et al. 2009). The term viaphyte has been suggested to refer to fungi that colonize leaves as endophytes and after leaf senescence or death they colonize woody parts of the tree. This ensures that the Xylaria are primary colonizers and can quickly dominate substrates (Nelson et al. 2020; Parfitt et al. 2010). These fungi also perform an important role as they change from endophytic colonizer to saprophytic wood degrader. Saprophytic fungi have a key role in the functioning of ecosystems since they are important in wood decomposition and contribute to the global recycling of carbon and other essential elements and minerals (Woodward 1998). Although little is known about their mechanisms of degradation, the large numbers of *Xylaria* found in the study area investigated here and their ability to cause significant biomass loss indicates they are important decomposers in the Amazon rainforest ecosystem.

The genus *Xylaria* has been reported as having special adaptations for survival including the production of melanin. This compound can be found in hyphae and fruiting bodies as well as in pseudosclerotial plates that form in wood substrates. These pseudosclerotial plates or zone lines can often completely enclose substrates providing a physical and chemical barrier that other competing fungi cannot breech. They can also be barriers that exclude excess moisture in very wet environments. The melanized morphological barriers appear to function well to allow these fungi to persist in abundance in this extreme environment. We know that melanin produced by other fungi can act to bind metal ions providing a metal ion sheath that functions to provide additional protection for the fungus (McDougall and Blanchette 1996; Rizzo et al. 1992; Toapanta Albán et al. 2022). This is also likely functioning in a similar manner for *Xylaria* species.

*Xylaria* are known to produce antimicrobial compounds (Canli et al. 2016; Jayasekara et al. 2022; Liu et al. 2008; Rakshith et al. 2020) that help the fungus survive interactions with other organisms (Elias et al. 2018). Many species have been studied for their use in the medical field and for human health applications. *Xylaria curta,* a species found in abundance and used in the decay studies of this paper has been found to have properties that are of great interest for medical application. The enzyme, Xylarinase, derived from *X. curta* is documented as having the ability to reduce blood clots (Meshram et al. 2016) and it has been reported to produce antibiotic compounds that could combat antibiotic resistant *Staphylococcus aureus* (Ramesh et al. 2012). The production of these antimicrobial compounds undoubtedly also plays an ecological role in aiding *Xylaria* species to maintain dominance among the large number of saprophytic fungi in the rainforest.

In our investigations, we used four different types of tropical woods with varying densities- (balsa, melina, saman, and moral) to assess the decay potential of three selected *Xylaria* species- two unknown *Xylaria* species (*Xylaria* 1 and 2) and *X. curta (Xylaria* 3). We chose to use multiple species of wood to better assess the degradation abilities on wood with different densities and extractives and to better understand the substrate range of *Xylariaceae* while interpreting their role in the nutrient cycling of the forest. The greatest loss of biomass was observed in balsa and melina wood, both of which had lower heartwood densities and since density is low they have low levels of heartwood extractives. This provides insight to the substantial amount of degradation that *Xylaria* species have and their capacity to cause significant decay in some tropical woods after a relatively short time. Even in woods with high density and increased amounts of heartwood extractives, the *Xylaria* species were able to cause appreciable amounts of degradation over the short incubation period of a few months in the laboratory. Results from a previous study in an Ecuadorian cloud forest, identified *X. curta* as a potentially significant decomposer (Thomas et al. 2016). These results support our conclusions and are also consistent with previous findings that *Xylaria* species have the ability to decompose lignin and carbohydrates in wood and have the ability to cause significant amounts of biomass loss (Nilsson et al. 1989; Osono 2020). In a laboratory wood decay study by Nilsson et al. (Nilsson et al. 1989) using *Xylaria hypoxylon*, approximately equal amounts of cellulose, hemicellulose and lignin were degraded. The *Xylaria* isolates we used in the study reported here caused significant mass loss in the agar microcosms in only a few months and their potential to cause mass loss in the rainforest is likely higher due to constant presence of abundant substrate, conducive temperatures for decay and high humidity that can be found throughout the year.

Our study demonstrated that the Ecuadorian Amazon *Xylaria* tested cause a soft rot type of wood decay. These species appear to have the ability to cause both Type 1 and Type 2 soft rot. Type 1 soft rot is characterized by biconical and cylindrical cavities within the secondary cell walls and Type 2 results in an erosion of the cell wall from the lumen to the middle lamella but leaves the middle lamella intact. In temperate areas of the world, Type 1 soft rot is commonly found in conifers and Type 2 in hardwoods (Eriksson et al. 1990). In the wood from the rainforest trees used in our experiments, Type 1 appears to occur in high extractive and higher-density tree species and Type 2 in lower density wood that has lower concentrations of extractives. Wood higher in extractives (saman and moral) showed less biomass loss compared to the woods that had fewer extractives (balsa and melina) which is consistent with other previously reported findings when testing rainforest tree wood using other species of fungi (Osono 2020; Toapanta Albán et al. 2022). Some studies suggest that *Xylaria* species are more efficient in degrading carbohydrates than lignin (Nghi et al. 2012; Osono 2020) and since the middle lamella is not degraded, they may have a preference for certain types of lignin over others (Blanchette et al. 1988; Eriksson et al. 1990; Sorvari et al. 1986). The inability to degrade the middle lamella in wood suggests these Ascomycota producing soft rot fungi may be missing some key degradative enzymes for lignin degradation. White rot Basidiomycota are able to effectively degrade lignin and all other cell wall components and have a wide variety of lignin degrading enzymes (Blanchette et al. 1997; del Cerro et al. 2021; Floudas et al. 2012; Riley et al. 2014; Schilling et al. 2020). Additional studies are needed to provide a more comprehensive view of the enzymatic and nonenzymatic processes used by tropical *Xylaria*. We would expect, based on the relatively low mass loss in high density woods over a short period, that given additional time substantially more degradation would occur. In tropical forests such as those at Yasuní National Park, decay takes place throughout the year, and this would allow the *Xylaria* species to cause degradation in wood from a diverse array of tree species, even those with high density and high extractives. The ability to colonize, tolerate, and degrade the extractive rich woods in tropical rainforests is likely the reason they are found so frequently in this unique environment.

Further research is needed to characterize *Xylaria* species, which are important for recycling woody biomass in the Amazon rainforest, and to better understand their role in healthy ecosystem functioning. Since so many unknown taxa were found in this study, taxonomic work should also be a priority to identify and name these new species. In addition, a better understanding of the enzymatic and nonenzymatic decay mechanisms used by *Xylaria* during wood degradation is needed. The remarkable diversity found in this study from surveys of just a single plot location suggests an enormous number of *Xylaria* species exists across the entire amazon rainforest and new research efforts are needed that focus on this important group of fungi.

### Supplementary Information

**Additional file 1.** Micrographs to illustrate the characteristics of Type 1 and Type 2 soft rot in a hardwood.

**Additional file 2.** List of sequences, and their accession numbers and ecuadorian Identification codes.

**Additional file 3.** Means percent of weight loss and standard deviations by treatment and wood type.

**Additional file 4.** Pairwise comparisons of mean percent of weight loss between inoculated treatments and the controls by wood type.

## Data Availability

All data are fully available without restriction in the manuscript and in the Additional files 2, 3, and 4.
