# Supplementary material for: Diverse Xylaria in the Ecuadorian Amazon and their mode of wood degradation
Source: Bot Stud. 2023 Oct 25;64:30. doi: 10.1186/s40529-023-00403-x (PMC10600087; doi:10.1186/s40529-023-00403-x)
Supplement: Supplementary file 1 — Additional file 1. Micrographs to illustrate the characteristics of Type 1 and Type 2 soft rot in a hardwood. [file 40529_2023_403_MOESM1_ESM.pdf]

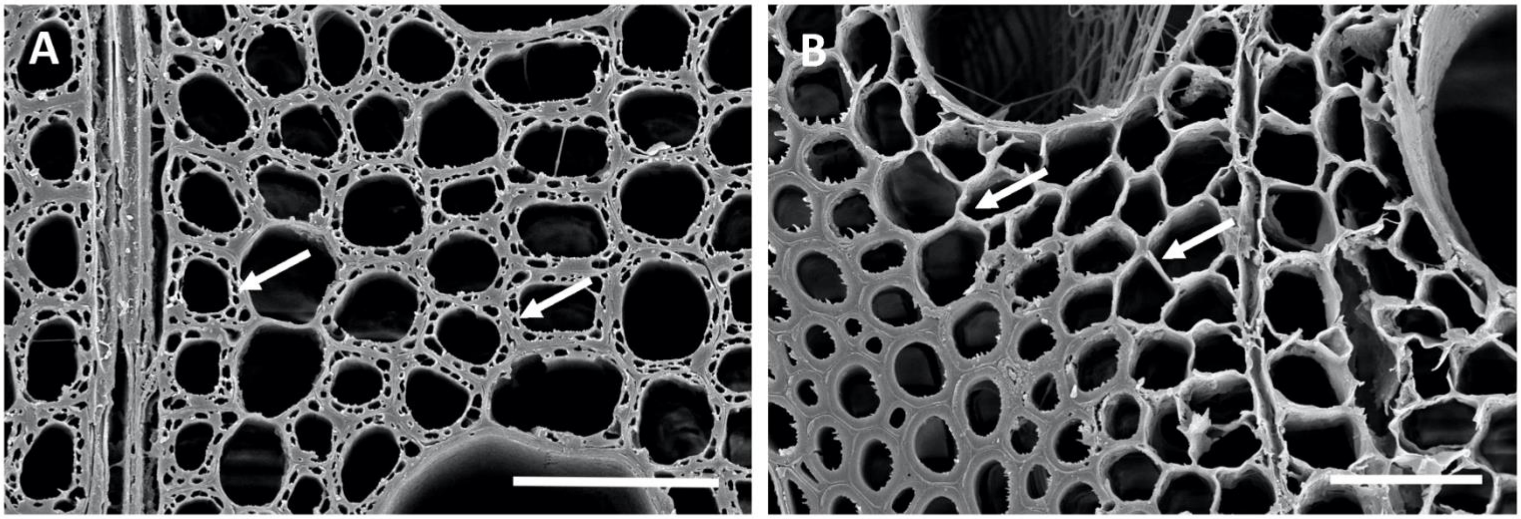

Supplemental Figure 1. Micrographs to illustrate the characteristics of Type 1 and Type 2 soft rot in a hardwood. A) Type 1 produces cavities within the secondary wall of wood cells (arrows). B) Type 2 erodes the secondary wall but does not degrade the compound middle lamella (arrows). Bar = 50 μm
