# Supplementary material for: Diverse Xylaria in the Ecuadorian Amazon and their mode of wood degradation
Source: Bot Stud. 2023 Oct 25;64:30. doi: 10.1186/s40529-023-00403-x (PMC10600087; doi:10.1186/s40529-023-00403-x)
Supplement: Supplementary file 3 — Additional file 3. Means percent of weight loss and standard deviations by treatment and wood type. [file 40529_2023_403_MOESM3_ESM.pdf]

Table 3 Means percent of weight loss and standard deviations by treatment and wood type.

	Fungus/Treatment	N	Mean Percent Loss	Std Deviation	P-value
Balsa	*Xylaria 1*	11	17.3	4.6	**<0.001**
	Xylaria 2	10	54.3	6.7	
	Xylaria curta	10	61	4.7	
	Control	10	4.6	2.4	
Melina	*Xylaria 1*	11	10	3.4	**<0.001**
	Xylaria 2	10	24.5	2.3	
	Xylaria curta	10	22.6	8.0	
	Control	10	3.2	3.6	
Saman	*Xylaria 1*	10	9.7	0.8	**<0.001**
	Xylaria 2	10	21	2.3	
	Xylaria curta	10	9.1	2.6	
	Control	11	5.4	3.3	
Moral	*Xylaria 1*	10	7.7	0.76	**<0.001**
	Xylaria 2	10	9.7	1.4	
	Xylaria curta	11	3.5	0.58	
	Control	8	2.5	0.53	

*P-values generated via ANOVA for the comparison of treatments within each wood type.
