# Supplementary material for: Diverse Xylaria in the Ecuadorian Amazon and their mode of wood degradation
Source: Bot Stud. 2023 Oct 25;64:30. doi: 10.1186/s40529-023-00403-x (PMC10600087; doi:10.1186/s40529-023-00403-x)
Supplement: Supplementary file 4 — Additional file 4. Pairwise comparisons of mean percent of weight loss between inoculated treatments and the controls by wood type. [file 40529_2023_403_MOESM4_ESM.pdf]

Table 4 Pairwise comparisons of mean percent of weight loss between inoculated treatments and the controls by wood type

	Comparison				
	Treatment	Mean Difference in Percent Loss	Lower Confidence Limit	Upper Confidence Limit	Adjusted p-value
Balsa	*Xylaria 1*	12.7	6.9	18.6	**<0.001**
	Xylaria 2	49.7	43.9	55.6	**<0.001**
	Xylaria curta	56.3	50.5	62.2	**<0.001**
Melina	*Xylaria 1*	6.8	1.2	12.4	**0.01**
	Xylaria 2	21.3	15.6	27.1	**<0.001**
	Xylaria curta	19.4	13.6	25.2	**<0.001**
Saman	*Xylaria 1*	4.3	1.4	7.1	**0.002**
	Xylaria 2	15.5	12.7	18.4	**<0.001**
	Xylaria curta	3.7	0.85	6.5	**0.006**
Moral	*Xylaria 1*	5.2	4.1	6.3	**<0.001**
	Xylaria 2	7.1	6.0	8.3	**<0.001**
	Xylaria curta	1.0	-0.1	2.1	0.1

*P-values and 95% confidence intervals generated via Tukey-Kramer Pairwise Comparison Method
